# Supplementary figures and images for: UDP-glucose dehydrogenase (UGDH) activity is suppressed by peroxide and promoted by PDGF in fibroblast-like synoviocytes: Evidence of a redox control mechanism
Source: PLoS One. 2022 Sep 15;17(9):e0274420. doi: 10.1371/journal.pone.0274420 (PMC9477357; doi:10.1371/journal.pone.0274420)

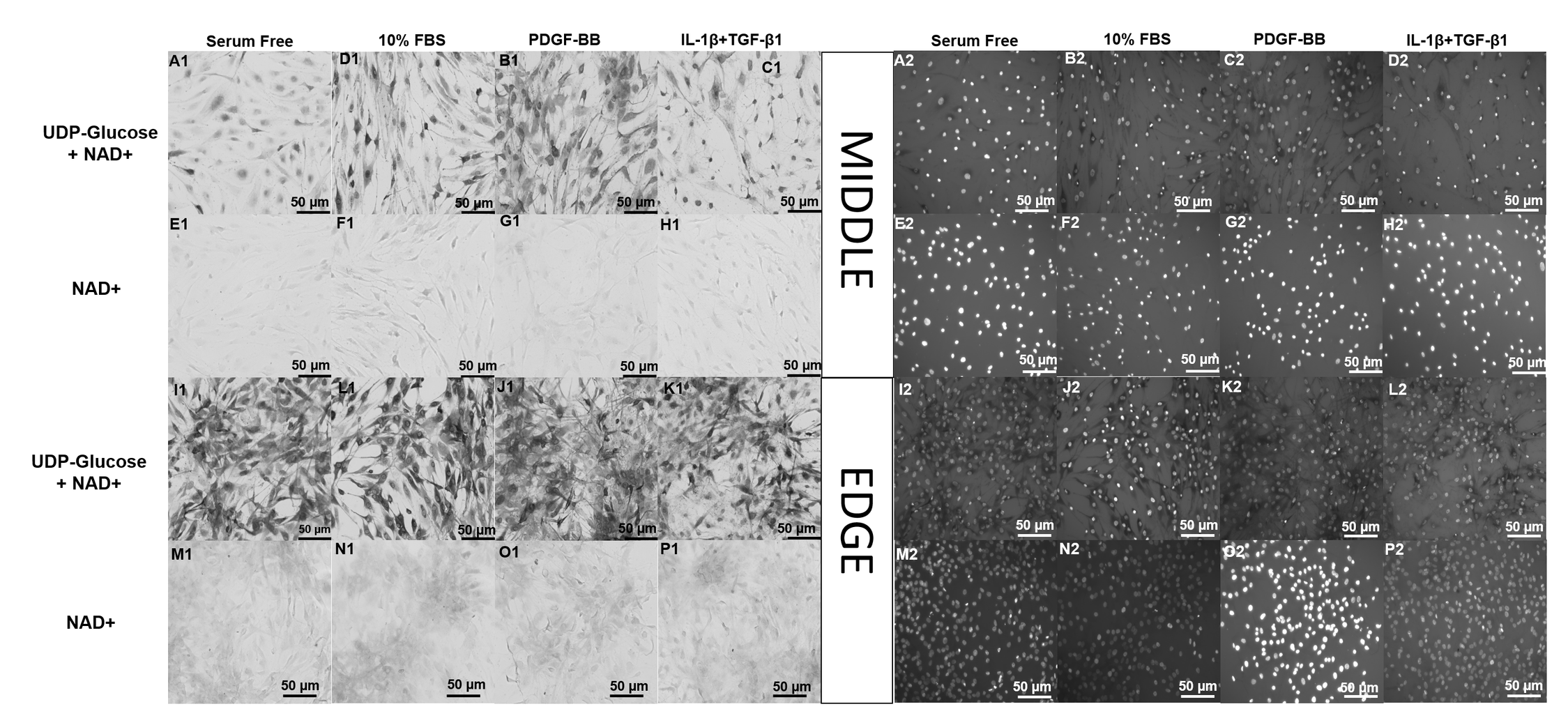

Supplement: S1 Fig — UGDH staining was higher at the culture well edge, where cells were aggregated compared to the middle of the well, therefore fields from the edge and middle of the well were averaged for the final quantitative result per culture well. Panels (A1-P1) show UGDH staining (grey scale) and (A2-P2) show Hoescht-stained nuclei of the same field. Scale bars: 50 μm. (TIF) [file pone.0274420.s001.tif]

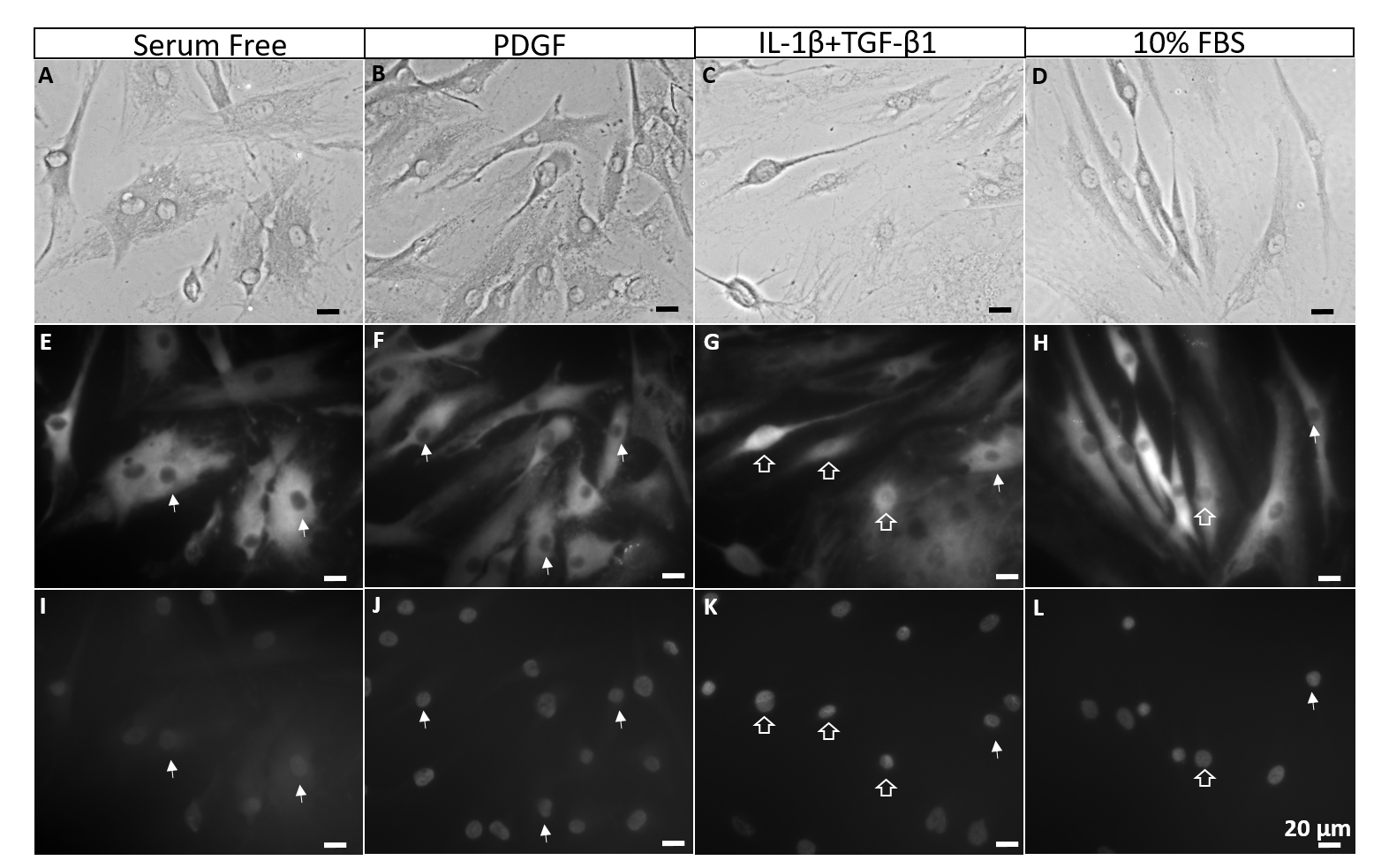

Supplement: S2 Fig — (A-D) Phase contrast and (E-H) matching epifluorescent images of UGDH immunostain and (I-L) DAPI-counterstained fluorescent nuclei (I-L) of FLS (P4) extracted from a synovial biopsy from a 23-year old male donor and cultured under different cytokine conditions as indicated, fixed in acetone, and immunostained for UGDH with red substrate detection which is fluorescent. Symbols: small white arrows: cytosolic UGDH immunostain; open arrows: detectable nuclear UGDH immunostain. Scale bars: 20 μm. (TIF) [file pone.0274420.s002.tif]

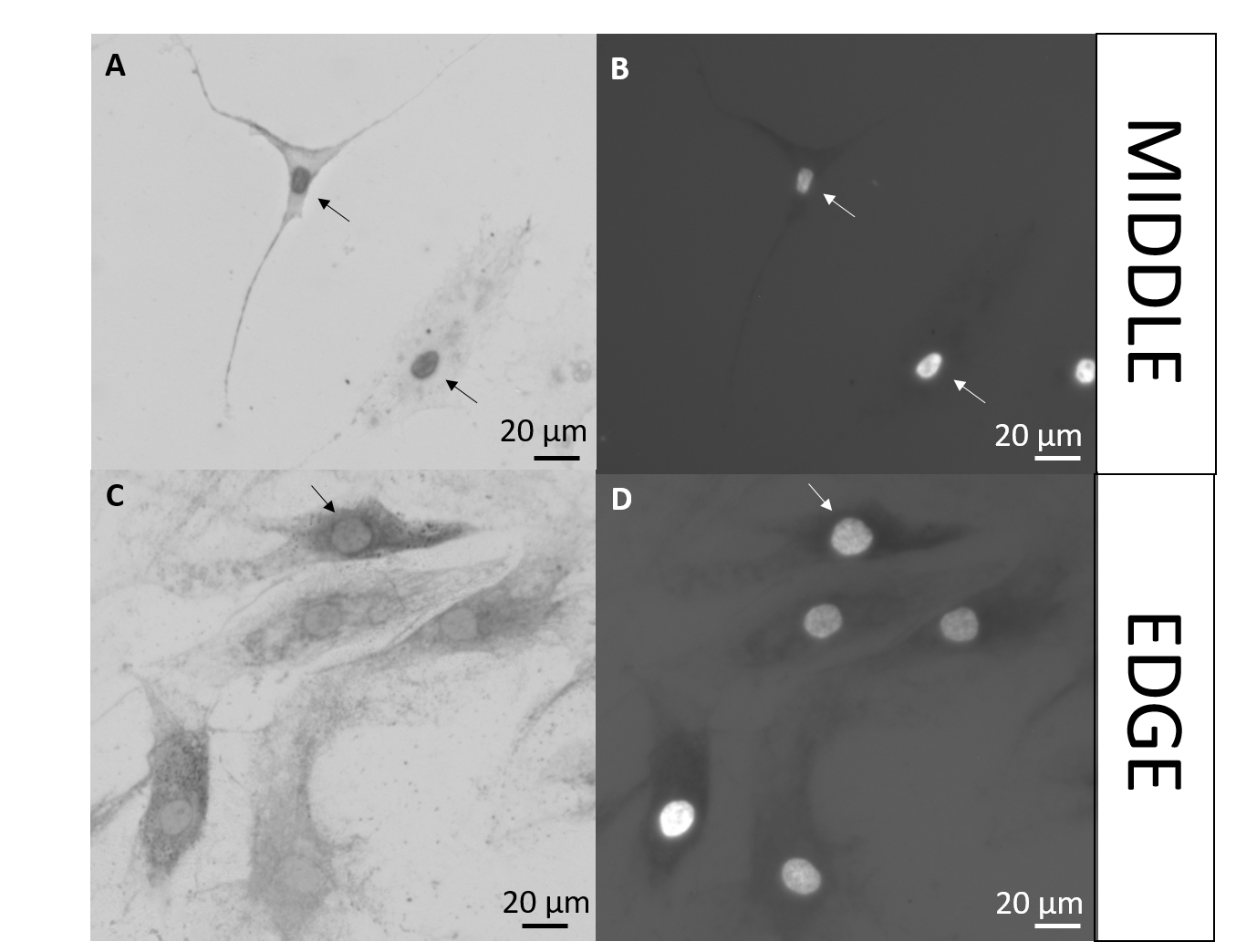

Supplement: S3 Fig — (A, B) UGDH enzyme histostaining was occasionally detected in the nuclear or perinuclear compartment in sub-confluent cells in the middle of the culture well, and (C, D) was mainly cytosolic in more confluent cells at the edge of the well. (A, C) bright field and (B, D) matching epifluorescence image of the same field showing Hoechst-stained nuclei of FLS stimulated here with 1 ng/mL each IL1β + TGF-β1 and stained for UGDH enzyme activity. Scale bars: 20 μm. (TIF) [file pone.0274420.s003.tif]

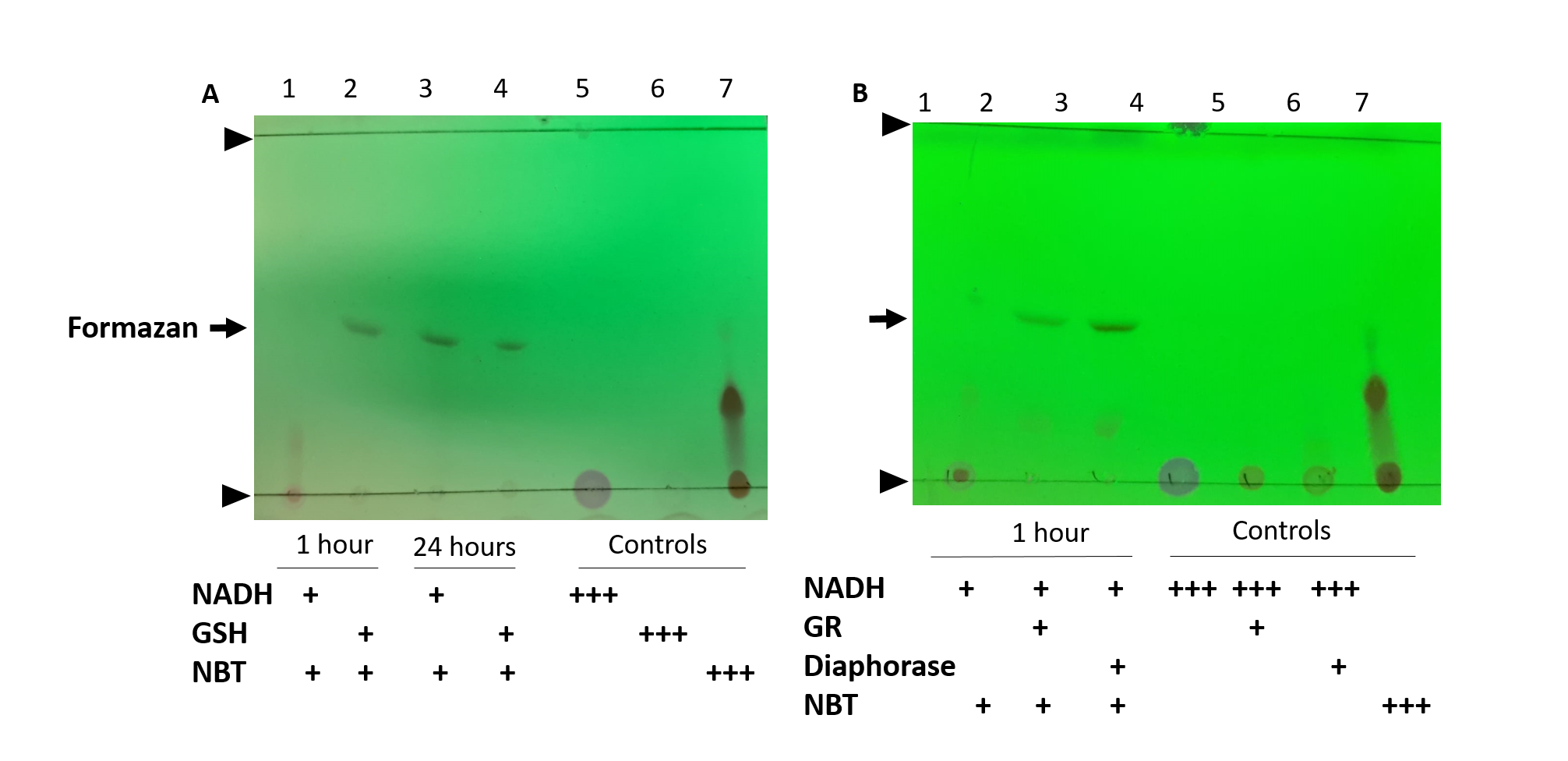

Supplement: S4 Fig — Arrowheads show the origin and final solvent front; UV light was absorbed in the TLC plate by NADH and NBT at the origin, and by diformazan at the arrow. Reduced formazan had an Rf value ranging from 0.42 to 0.44 [38] depending on whether the lane was in the middle or edge of the TLC plate. In the absence of enzyme, GSH reduced NBT after 60 minutes but NADH only reduced NBT after 24 hours. Diaphorase or GR was necessary and sufficient for NADH to reduce NBT within 1 hour. See (S1 File) for original TLC plate images. (TIF) [file pone.0274420.s004.tif]

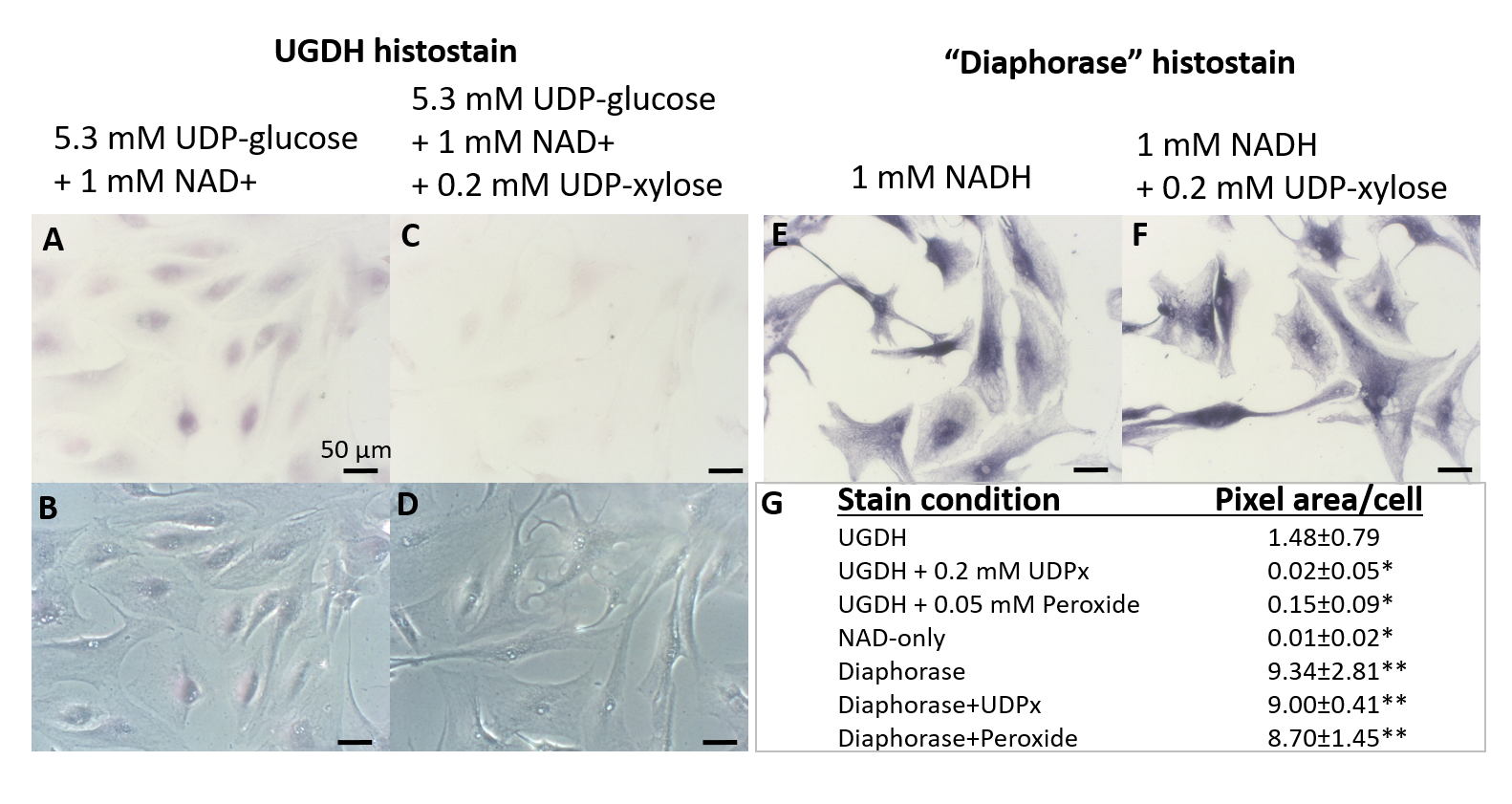

Supplement: S5 Fig — Cells were from a 23-year old male donor (passage 4). FLS stained for UGDH (A: bright field; B: phase contrast) or UGDH with UDP-xylose (C: bright field; D: phase contrast), diaphorase (E), or diaphorase with UDP-xylose (F). In panel (G), quantitative histomorphometry was used to measure the relative staining intensity in the absence or presence of UDP-xylose or peroxide. Symbols: *p<0.01 vs UGDH; ** p<0.0001 vs UGDH. Scale bars: 50 μm. (TIF) [file pone.0274420.s005.tif]

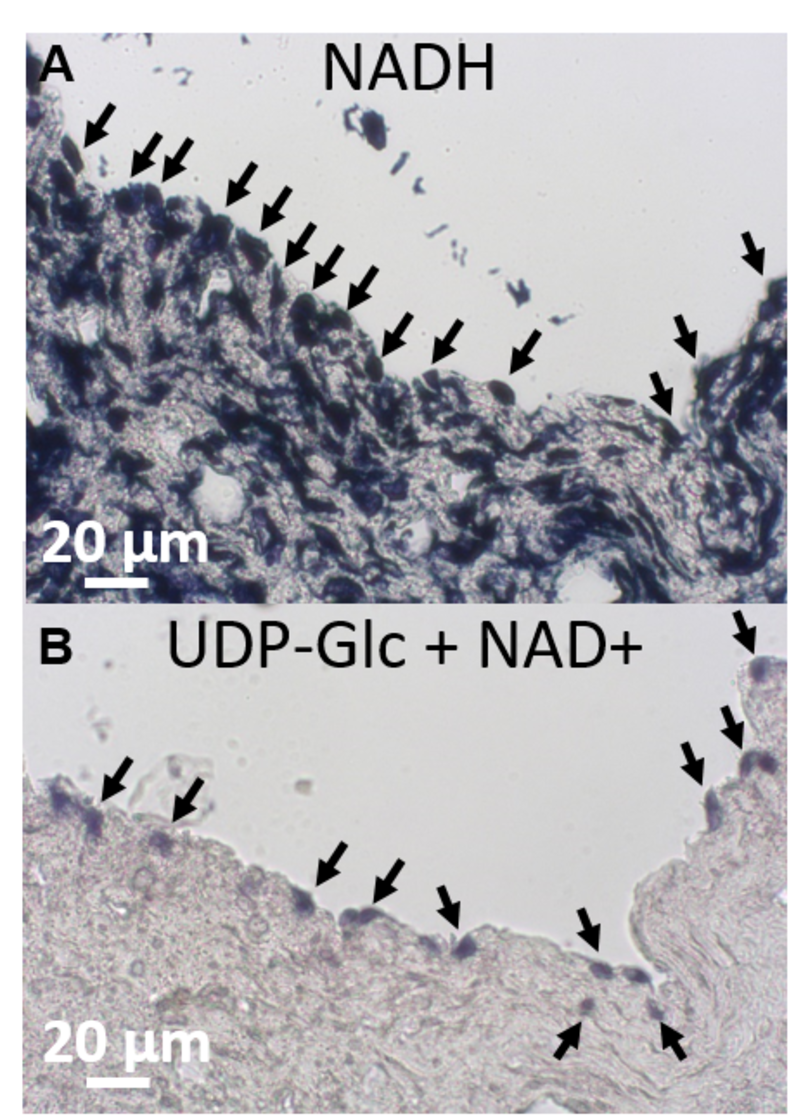

Supplement: S6 Fig — Serial infrapatellar synovial membrane cryosections from male rabbit 386 were enzyme histostained for (A) diaphorase/GR, and (B) UGDH activity. Note that some UGDH+ cells are displaced to the sub-synovium. (TIF) [file pone.0274420.s006.tif]
