# Supplementary material for: UDP-glucose dehydrogenase (UGDH) activity is suppressed by peroxide and promoted by PDGF in fibroblast-like synoviocytes: Evidence of a redox control mechanism
Source: PLoS One. 2022 Sep 15;17(9):e0274420. doi: 10.1371/journal.pone.0274420 (PMC9477357; doi:10.1371/journal.pone.0274420)
Supplement: S1 Table — (DOCX) [file pone.0274420.s007.docx]

Supplementary Table 1: Inclusion/exclusion criteria for human subjects recruited to the study

| **Inclusion Criteria** |
| --- |
| - Subjects aged 19 to 40 (male and female) |
| - Knee pain (at least 5 on a scale of 0 to 10, 10 being extreme pain) |
| - Good health |
| - Scheduled to undergo knee arthroscopy for knee injury |
| - Voluntarily cease anti-inflammatory medication 1 week prior to surgery. |
| - Are able to provide informed consent |
| - Are willing to provide a synovial biopsy and citrated blood plasma sample. |
|  |
| **Exclusion criteria** |
| - Prior local corticosteroid injection in the knee, or oral corticosteroids within 1 week. |
| - Prior treatment with bone-altering medications (teriparatide/PTHrP, bisphosphonates/alendronate, estrogen replacement) |
| - Hyaluronic acid knee injection within 6 months prior |
| - Chronic inflammatory disorders or poly-arthritis (rheumatoid arthritis) |
| - Knee infection or other infection |
| - Hereditary connective tissue diseases |
| - Endocrine disorders |
| - Hemophilia or history of multiple blood transfusions |
| - Pregnancy |
| - Neuromuscular, neural, or circulatory disorder affecting the injured limb |
| - Known infectious disease (HIV, hepatitis A, B, or C) |
| - Incapable of making informed decision |
