# Supplementary material for: UDP-glucose dehydrogenase (UGDH) activity is suppressed by peroxide and promoted by PDGF in fibroblast-like synoviocytes: Evidence of a redox control mechanism
Source: PLoS One. 2022 Sep 15;17(9):e0274420. doi: 10.1371/journal.pone.0274420 (PMC9477357; doi:10.1371/journal.pone.0274420)
Supplement: S1 File — (PDF) [file pone.0274420.s008.pdf]

S1 File: Supporting information for Figure S4 including lane contents and original TLC images

Panel A

Lane	Content
1.	NADH + NBT, after 1 hour of incubation
2.	GSH + NBT, after 1 hour of incubation
3.	NADH + NBT, after 24 hours of incubation
4.	GSH + NBT, after 24 hours of incubation
5.	NADH-only (negative control)
6.	GSH-only (negative control)
7.	NBT-only in excess (negative control)

Panel B

Lane	Content
1.	NADH + NBT, after 1 hour of incubation
2.	NADH + NBT + Glutathione Reductase, after 1 hour of incubation
3.	NADH + NBT + Diaphorase, after 1 hour of incubation
4.	NADH-only (negative control)
5.	NADH + Glutathione Reductase, after 1 hour of incubation (negative control)
6.	NADH + Diaphorase, after 1 hour of incubation (negative control)
7.	NBT-only in excess (negative control)

Bottom solid line: area where reagents were spotted

Top solid line: solvent front

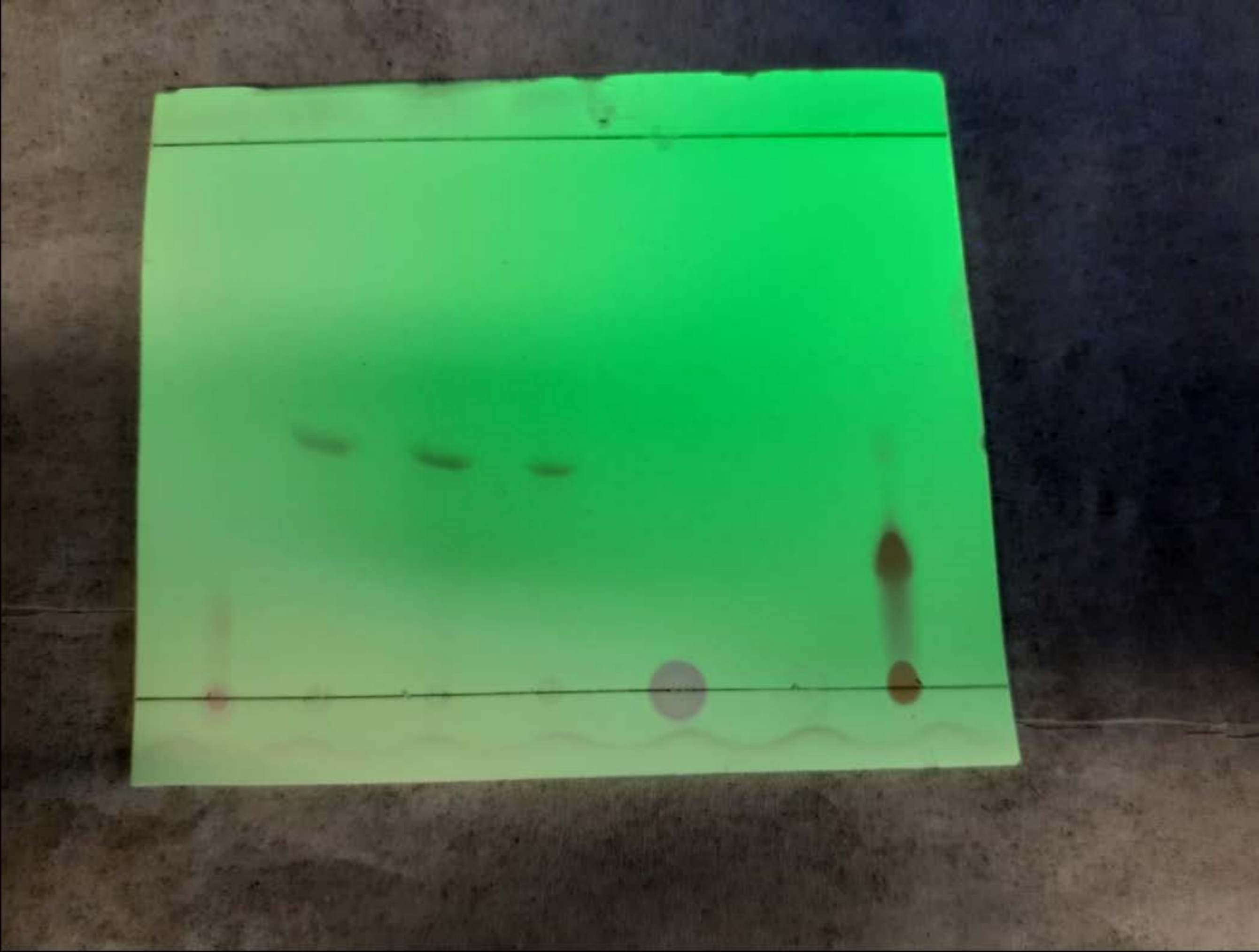

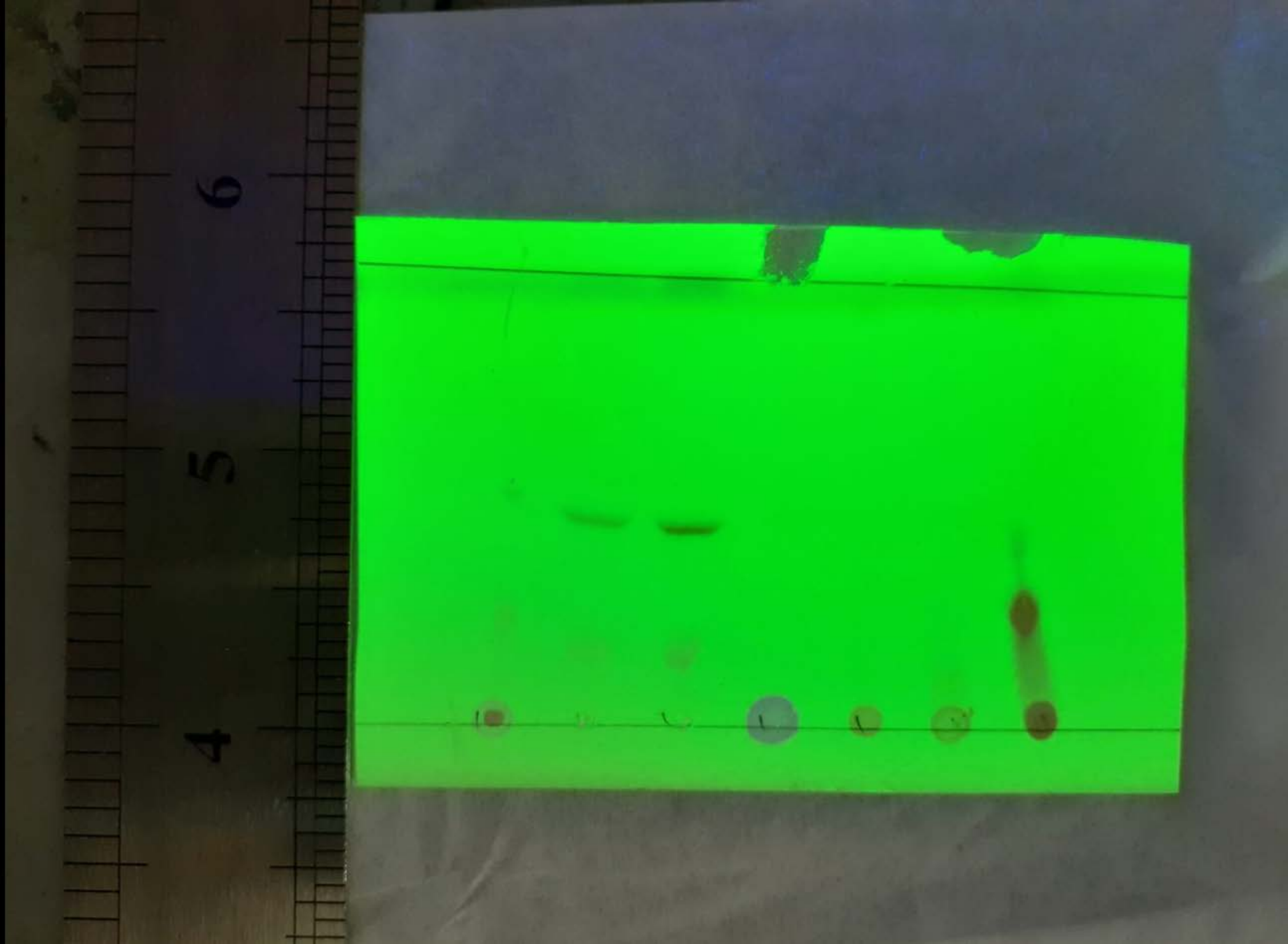
6
5
4
