# Supplementary material for: UDP-glucose dehydrogenase (UGDH) activity is suppressed by peroxide and promoted by PDGF in fibroblast-like synoviocytes: Evidence of a redox control mechanism
Source: PLoS One. 2022 Sep 15;17(9):e0274420. doi: 10.1371/journal.pone.0274420 (PMC9477357; doi:10.1371/journal.pone.0274420)
Supplement: Attachment — Submitted filename: Response to Reviews.docx [file pone.0274420.s010.docx]

**From:** em.pone.0.7d58a9.2b49b5e9@editorialmanager.com <em.pone.0.7d58a9.2b49b5e9@editorialmanager.com> on behalf of PLOS ONE <em@editorialmanager.com>
**Sent:** Monday, August 15, 2022 1:06 AM
**To:** Caroline Hoemann <choemann@gmu.edu>
**Subject:** PLOS ONE Decision: Revision required [PONE-D-22-03359R1] - [EMID:e2c1958328bb275f]

PONE-D-22-03359R1

UDP-glucose dehydrogenase (UGDH) activity is suppressed by peroxide and promoted by PDGF, diaphorase and glutathione reductase in fibroblast-like synoviocytes

PLOS ONE

Dear Dr. Caroline D Hoemann,

Thank you for submitting your manuscript to PLOS ONE. After careful consideration, we feel that it has merit but does not fully meet PLOS ONE’s publication criteria as it currently stands. Therefore, we invite you to submit a revised version of the manuscript that addresses the points raised during the review process.

Please submit your revised manuscript by November 13, 2022. If you will need more time than this to complete your revisions, please reply to this message or contact the journal office at [plosone@plos.org](mailto:plosone@plos.org). When you're ready to submit your revision, log on to https://www.editorialmanager.com/pone/ and select the 'Submissions Needing Revision' folder to locate your manuscript file.

Please include the following items when submitting your revised manuscript:

- A rebuttal letter that responds to each point raised by the academic editor and reviewer(s). You should upload this letter as a separate file labeled 'Response to Reviewers'.
- A marked-up copy of your manuscript that highlights changes made to the original version. You should upload this as a separate file labeled 'Revised Manuscript with Track Changes'.
- An unmarked version of your revised paper without tracked changes. You should upload this as a separate file labeled 'Manuscript'.

If you would like to make changes to your financial disclosure, please include your updated statement in your cover letter. Guidelines for resubmitting your figure files are available below the reviewer comments at the end of this letter.

If applicable, we recommend that you deposit your laboratory protocols in protocols.io to enhance the reproducibility of your results. Protocols.io assigns your protocol its own identifier (DOI) so that it can be cited independently in the future. For instructions see: [https://journals.plos.org/plosone/s/submission-guidelines#loc-laboratory-protocols](https://secure-web.cisco.com/1Iyp0cD-G2N2WTHugWo9_Er8_iFXZ6VSD_XBtkubGd3uAPR4tXqUY6J8hMQk9N97r6TtgZxuMB54ofDZwz7NE3njEFJbXzOOX1QXVWtG7FIfSOWRgygEB3JvJdR3m7XMCVgQiIcbxAnH1cMCvZ_ZIttmYPEgkNK8f79fTTzfePIvaZg6-m6qlpr3oaqRf9R0lYSPT1IM8sLWMiUImfVdf9BKhr1BKkgr2l5a3yjPt3a-NraqxHap8L7-Npk4qF_V-S3rP_bhkd3mNBpPD00S0BQGFnKcjKZ02lpSWscdKSwbEYKVFP86CY-G5HbTagdiz3zyf_Uj5z1HWEUA4a2y-fHCiyMQxyM8w0qI-fc2TxwugPpJh4qyCVxei12T0wa7OaIRZRObhgiopcKltjMKMgWbiUetYCeWAQXWtMYaVSgDiXKqjUWOBj42BU8susDmU/https%3A%2F%2Fjournals.plos.org%2Fplosone%2Fs%2Fsubmission-guidelines%23loc-laboratory-protocols). Additionally, PLOS ONE offers an option for publishing peer-reviewed Lab Protocol articles, which describe protocols hosted on protocols.io. Read more information on sharing protocols at [https://plos.org/protocols?utm_medium=editorial-email&utm_source=authorletters&utm_campaign=protocols](https://secure-web.cisco.com/1jbnPV3tLnyU0SDeE96oDKwPC-k5g8Hj7PVQBi5PgvuQgvVz81o7rukZyVXZrbgv7c_I2CPOVfPJpqM96oLpZRz83v3pMOR1YCCa7fb_msWGqinXCRx3XdTCgXuH0WLiXTA_fZQoYXoM7O8bOq4Sdb_o4Vx8YcC3gEx0l3oO_GkS1ZIE-q-0nIeDAx_WMMH6ZXQnACg0WuKBtTs55x7ArJAk5jrSjx-WxSKlk9Ho3Bu7ru5Dap_coAh5sR3Vj7J6ifYYIbzKpwkpOVALOp5x-k9G85buSsMCPmlRGZUGBTCeAGqqehd-KqTNlxsDN176lNCQ1rheUg72J8o8HRyOjiDpLUkga4Cw_qTmRltV3_YcR-ogiSWahGPzfWN1uGBFrl41DFmV7Y7IjmiywBqfah4GNyQ15JnqHsZ3v_7NoRV1MEJ7jOc3Nxw8k4glrsiBq/https%3A%2F%2Fplos.org%2Fprotocols%3Futm_medium%3Deditorial-email%26utm_source%3Dauthorletters%26utm_campaign%3Dprotocols).

We look forward to receiving your revised manuscript.

Kind regards,

Abdelwahab Omri, Pharm B, Ph.D, Laurentian University  Canada
Academic Editor
PLOS ONE

[Note: HTML markup is below. Please do not edit.]

Reviewers' comments:

Reviewer's Responses to Questions

**Comments to the Author**

1. If the authors have adequately addressed your comments raised in a previous round of review and you feel that this manuscript is now acceptable for publication, you may indicate that here to bypass the “Comments to the Author” section, enter your conflict of interest statement in the “Confidential to Editor” section, and submit your "Accept" recommendation.

Reviewer #1: All comments have been addressed

Reviewer #2: (No Response)

2. Is the manuscript technically sound, and do the data support the conclusions?

The manuscript must describe a technically sound piece of scientific research with data that supports the conclusions. Experiments must have been conducted rigorously, with appropriate controls, replication, and sample sizes. The conclusions must be drawn appropriately based on the data presented.

Reviewer #1: Yes

Reviewer #2: Partly

3. Has the statistical analysis been performed appropriately and rigorously?

Reviewer #1: Yes

Reviewer #2: Yes

4. Have the authors made all data underlying the findings in their manuscript fully available?

The [PLOS Data policy](http://secure-web.cisco.com/1Qn3M04QElGNhkV3KaD5SdbBC2cEfQtHbn6fuumYgd3tHp9Tp0kCbn_m2j_mEJ1GhTuIvPvqR-KcLzPLCpRNg7846gkT5REiYbYXliGdSWA2TWVVM02wEHESK3VVY63Tv9D1K5jCJEMWhz-i9zWaj958OYmYTO-aZNMP7nQljjIVYmAjV0t_wAUiCX393ImXgqRq7IopQSfpn4TCqkf544PcIrYGTbVd6_rdQCDHutMujSUv0lf5VLtGAtRCDaK4V-1sYZY7TRo7v8_PM2uc3rEvOqx4BgbCpgaYtExiCq8smdMrgfAH8ND8yMQxixN2YcFYPFRbZUhcMSDvlVUgKeglQNWxayFD8Sf_QAckoByj3wsLk6ViYKQB3xUE4GeZMl2yE-eifH-hMbOf1AGGCyViTGJmXqZomlHotA-LC_Uq2q-y3mGFoxGVrm-eJ-VIRan1nznrt5L1K_pDJRvp0-A/http%3A%2F%2Fwww.plosone.org%2Fstatic%2Fpolicies.action%23sharing) requires authors to make all data underlying the findings described in their manuscript fully available without restriction, with rare exception (please refer to the Data Availability Statement in the manuscript PDF file). The data should be provided as part of the manuscript or its supporting information, or deposited to a public repository. For example, in addition to summary statistics, the data points behind means, medians and variance measures should be available. If there are restrictions on publicly sharing data—e.g. participant privacy or use of data from a third party—those must be specified.

Reviewer #1: Yes

Reviewer #2: Yes

5. Is the manuscript presented in an intelligible fashion and written in standard English?

PLOS ONE does not copyedit accepted manuscripts, so the language in submitted articles must be clear, correct, and unambiguous. Any typographical or grammatical errors should be corrected at revision, so please note any specific errors here.

Reviewer #1: Yes

Reviewer #2: Yes

6. Review Comments to the Author

Please use the space provided to explain your answers to the questions above. You may also include additional comments for the author, including concerns about dual publication, research ethics, or publication ethics. (Please upload your review as an attachment if it exceeds 20,000 characters)

Reviewer #1: the authors have greatly improved the text by clearly responding point by point to all the comments raised in the previous version of the manuscript

Reviewer #2: In this revised manuscript, the authors have strongly addressed the majority of the prior critiques. The manuscript reports and interprets intriguing biological phenomena related to arthritis and its associated pathologies utilizing a combination of human specimens and an elegant rabbit model of arthritic injury. A number of technical issues have been thoroughly addressed and discussed in a manner that will have significant value to researchers in a variety of related fields.

The key lingering concern is that some of the biological interpretations emphasized by the authors have not been explicitly validated by the authors. The point of concern is not that the speculation about peroxide oxidation of an active site cysteine, and its resolution by redox buffering proteins like glutathione reductase and diaphorase, is not feasible, valid, or supported by the data they have generated, but rather that some of the interpretations are subjective and there are alternatives that are not fully considered by the authors as indicated in the prior review. The title of the manuscript immediately prompts the expectation that the biological interpretation particularly surrounding redox potential and driving sources of redox buffering is the experimental focus of the paper. However, the interpretation actually relies on a selection of published results that have not been reproduced by the authors. Simply softening the title would mitigate this expectation and better represent the impact of the manuscript. This would be an adequate solution to the biological interpretation concerns.

Response: we thank the reviewer for these comments and have modified the title of our paper.

Original title:

UDP-glucose dehydrogenase (UGDH) activity is suppressed by peroxide and promoted by PDGF, diaphorase and glutathione reductase in fibroblast-like synoviocytes

Modified title:

UDP-glucose dehydrogenase (UGDH) activity is suppressed by peroxide and promoted by PDGF in fibroblast-like synoviocytes: evidence of a redox control mechanism

The authors made the following observation in their response: “…we wish to draw your attention to the supportive comments of Reviewer 1, who agreed to disclose his identity in a transparent review process. Dr. Passi is an internationally-recognized expert in the field of hyaluronic acid synthesis and UGDH regulation, and his comments show that he finds merit in our findings.”

Though it does not influence this review or the determination of the manuscript’s significance, this statement offers an opportunity for a professional development comment. Three implications derive from this statement and how it is worded: 1) that revelation of reviewer identity indicates review transparency and reviewer competency; 2) that identity of the reviewer shows research merit; and 3) that failure to reveal identity indicates lack of competence or integrity - or even support - in critical evaluation of scientific publication. PLOS One is one of several progressive journals that offer the option for reviewers to reveal their identity. There are many reasons, both positive and negative, for reviewers to reveal or conceal identity. Many of the most qualified scientific reviewers will decline to review if their identity is compulsorily made public. Competency of the reviewer can be presumed by the quality of the review comments and the reviewer’s acceptance of the opportunity to review the response. It is easy to deflect a critical review by denigrating the character or academic record of an experienced reviewer if compelled to reveal identity, for example, rather than accepting the comments and responding to them in good faith. Moreover, journal editors vet the quality and expertise of scientific reviewers before inviting them to review a manuscript. The statement made by the authors can be interpreted as an accusation to the editor and the reviewer that there is a lack of expertise in the review since the reviewer is anonymous to the author. The author now has the choice to submit to a range of journals with policies that do not permit anonymity if they do not have faith in anonymous review.

Response: We sincerely apologize for any unintended affront, and did not wish for our comment to be negatively construed by any means. In some peer-reviewed operating grant committees, reviewers are asked to declare their level of expertise, all for the sake of aiding the peer review process. We would like to re-affirm our appreciation for your thoughtful review of our paper and the professional editorship of the PLoS One journal, and acknowledge that the debate of transparent, blinded, and double-blinded peer review is outside the scope of our paper.

7. PLOS authors have the option to publish the peer review history of their article ([what does this mean?](https://secure-web.cisco.com/1Gzqn-XW6Qv6_Gc14WW1qC7vk8pJGSC23npYDDpn2K6NGGUX6yP-ngjrWRKo21cw8QsljepyTxc-tm13xddQVuR6Reoss0L60GOgd0aOagxacuXS0AijnjLNwOVFfWgQknhuEMnS1VKUqilOVQEVwZQDr5c-mLskX88h7laS-FCIqVsDPZadv3NPPWykz9AFhUm23ZFFyOEmDTdDW9q6ALEc89JdJQjZQe11CEkFWKBvR5v05hEw0pSG2jfVM3ycvjPzURLyls4Euyh8DwJYfnnOEn2cVDpiBv0ckz2AmuPWNrw4e_EvjPYYhwdE2tvYYVNqI9UnfPtrITNS7qiOyrXV2nimoDdlAomgTTu4bYeTbfqHtxfzFbrpJ31Im93K4QaTg_nobH3zlQ_iCh5RFS0eLUwGE99pVmWVol1EYTf1gZIRoa0C0iY84Z17DQhNz/https%3A%2F%2Fjournals.plos.org%2Fplosone%2Fs%2Feditorial-and-peer-review-process%23loc-peer-review-history)). If published, this will include your full peer review and any attached files.

Response: We agree to publish the peer review history of our paper.

If you choose “no”, your identity will remain anonymous but your review may still be made public.

**Do you want your identity to be public for this peer review?** For information about this choice, including consent withdrawal, please see our [Privacy Policy](https://secure-web.cisco.com/1E6CsUQzlgf8JQP10OKuvz_k8x0j1HiGm2H7LU4pgZc2JTUBQdJDnq8e_xLOoxYkUpqiqZJpwJ9S3dHbS7W5Kyc65GN2MMSekjBuOztk5fp0SOuvQrxOXjRBIcItpPFphhDyH4kTVnRovripr2vnhQx7wUcuGs0KQtjXh-giVokbQxH3eScqXgDZb2ubDo2M8Q9v3UYnm4JZLIpyQYba7u1OKmtGCUfvW7lhfHaKVcWza7qpFq25OJSBT-bers32d4qVsZTsGyrGyR0FJAFEN8sa0ar7y0W40bmlrSNm6GDIOqdYVoA0fRAZsUCD5OG_g7-vFWauIgy4yrKcU7WRDxg3SW_JCsFya-S4flYLP_vilh5PBmkmLQ0Vm8Da1gNafoazpTr6ZgOydzq3t1eYREeVHEAKL5JVVqQlm88KmAuLegjR8ESZa1jgYPeSUqjmMYHh4ARvcDym7WDOHyD7VIA/https%3A%2F%2Fwww.plos.org%2Fprivacy-policy).

Reviewer #1: **Yes:** alberto passi

Reviewer #2: No

[NOTE: If reviewer comments were submitted as an attachment file, they will be attached to this email and accessible via the submission site. Please log into your account, locate the manuscript record, and check for the action link "View Attachments". If this link does not appear, there are no attachment files.]

While revising your submission, please upload your figure files to the Preflight Analysis and Conversion Engine (PACE) digital diagnostic tool, [https://pacev2.apexcovantage.com/](https://secure-web.cisco.com/1h_wQoUNZ9qKK2gJcwLTvsGV1-6_3YGlKOoseelfJSkuor8ed8FkrLrRGkeMxdnYfQCRcxC79cFchWePjdzP7Up2BmhwyU1rJptYTB2yrbrl_IL-miq8KL6v-6cuc55lEV-8JVOdPHxPaLK4JhyUtHNvs9jzeLjAecWyVvlZi6-xDHZCtY0513unaPvakwst2a0jp1hcisTUCMAQ7GduAEPlmdfYpxPZpXeeFxdqq5eIFhOxDyzKXDKm1USJbwzKmO3FSqXblObpvweCQxoZBnM0wEGvteA5cl6sz3E6EHIhJkk98EzhHJmUDep-Ww5x3Z8EQcaGxyfSX71ZIVc_m9d509NFLUUbq4voWzED-rYveuZQkYzIk7wL7u2OwKvQyWDAm4wVYK0K2Qg2-P93pgN9dw2U67bY7yy4KEpkMsBdmR72CAkYg9m9xijzCmJUD/https%3A%2F%2Fpacev2.apexcovantage.com%2F). PACE helps ensure that figures meet PLOS requirements. To use PACE, you must first register as a user. Registration is free. Then, login and navigate to the UPLOAD tab, where you will find detailed instructions on how to use the tool. If you encounter any issues or have any questions when using PACE, please email PLOS at [figures@plos.org](mailto:figures@plos.org). Please note that Supporting Information files do not need this step.

*In compliance with data protection regulations, you may request that we remove your personal registration details at any time.* [*(Remove my information/details)*](https://secure-web.cisco.com/1bUmoT3ls0m60R87gsYNSfSgSIaoLbtx8s6M7ZIf7Q1cFBVv3NwOIW_s5JANtsl4u1GD3Qho5GnHwOsTsF93EYMb-Vj8QGzXYgqgJXv8wSVXBtgj2oZ8otXnAsw4OqLVCkAnmyznvmTzSajoQWCL8WdbNukwnnsWHnZ1bQ1wTj-ZFw4A9xhRHdCfDd69U-dnKlJuFSrSpFePOGTUt41w9PmKxg2dIjtHUI80wYzjQz_b2MxMi3Ev0_l8IbnojeCnasUNktPOKKmpsfI5Y-G9w9cMeP4t7XxBusXwNWMp2IP1xpqPvGge5nNi7lilThUkI-OXXO0ORMzgQi7nyN5-92lkeLEPx53n-O-39I4yEGcZlC7M70kfKoD9ZjtzCfT-6YSGp-4DOUIIitRzSifsYL6v_bfxIe6qWsrsIj8tD9JqJwOBSdNH3pa8iTYk052Ko/https%3A%2F%2Fwww.editorialmanager.com%2Fpone%2Flogin.asp%3Fa%3Dr)*. Please contact the publication office if you have any questions.*
